# Contrasting Coastal Environments Are Associated With Ecotype‐Specific Leaf Polyphenolic Profiles and Bioactivity in *Corema album* subsp. *album*

**DOI:** 10.1002/cbdv.71705

**Published:** 2026-09-20

**Authors:** Eliana Fernandes, Riccardo Trentin, Maria João Rodrigues, Luísa Custódio

**Affiliations:** ^1^ Centre of Marine Sciences (CCMAR/CIMAR LA), Faculty of Sciences and Technology University of Algarve Faro Portugal; ^2^ Department of Biology University of Padova Padova Italy

**Keywords:** antioxidants, *Corema album*, cytotoxicity, ecotypes, LC–MS metabolomics

## Abstract

*Corema album* subsp. *album* is an endemic coastal shrub adapted to the harsh Mediterranean–Atlantic interface, where populations experience strong microclimatic variation. Understanding how such environmental pressures contribute to shaping its metabolic profiles is key to supporting conservation and sustainable valorization strategies based on the responsible use of renewable plant biomass, while reducing pressure on wild populations. This study compared the phytochemical composition and bioactivities of leaf extracts from two ecotypes collected in southern Portugal. Leaves were selected as a renewable and understudied organ with antioxidant and pharmacological potential. Both ecotypes were rich in (poly)phenols, mainly flavonoids, along with phenolic acids, triterpenoids, and representatives of coumarin and anthraquinone, and exhibited strong antioxidant capacity. The Vila Real de Santo António ecotype (CL1), exposed to higher solar radiation and drought, accumulated more total (poly)phenolics, hydroxycinnamic acids, and flavonoids, displaying stronger copper‐chelating and cytotoxic activities. Conversely, the Aljezur ecotype (CL2), from a cooler and more humid habitat, was richer in flavonols, hydroxybenzoic acids, and organic acids, supporting efficient redox‐based antioxidant protection with lower cytotoxicity. These ecotypic differences reveal adaptive metabolic strategies that enhance stress resilience, highlighting *C. album* leaves as a promising renewable source of bioactive compounds for functional and sustainable health applications.

## Graphical abstract

Leaves of two *Corema album* subsp. *album* ecotypes from two contrasting coastal microclimates were analysed using mineral profiling, (poly)phenolic assays, and LC–MS metabolomics. Ecotype‐specific metabolic strategies were revealed, linking environmental stress to antioxidant and cytotoxic activities. These findings highlight leaves as a renewable bioresource for climate‐smart conservation and functional health applications.

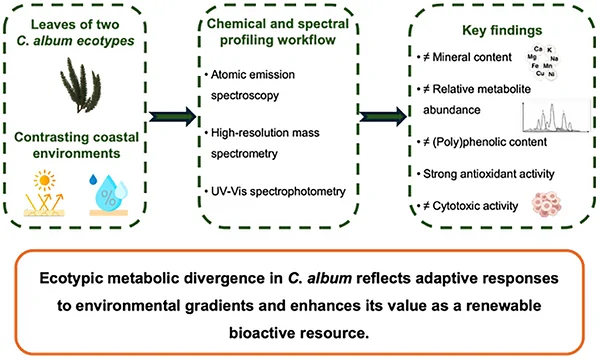

## 1 Introduction

The genus *Corema* (family Ericaceae) comprises two known species: *Corema conradii* (Torr.) Torr. ex Loud., native to the eastern coast of North America [1], and *Corema album* (L.) D. Don, which includes two subspecies: *C. album* subsp. *azoricum* Pinto da Silva, a recently discovered taxon, found in the Azores archipelago, and *C. album* subsp. *album*, distributed along the Atlantic coasts of the Iberian Peninsula [2, 3].

*Corema album* subsp*. album* occupies coastal habitats such as dunes, rocky cliffs, and pine forests, where it is exposed to extreme temperatures, salinity, wind, and nutrient‐poor soils [3, 4, 5, 6, 7, 8]. Along the Portuguese coast, populations are most abundant in the central–northern (Nazaré–Ovar) and southwestern (Sines–Tróia) regions, while more fragmented groups persist in areas such as the Southwest Alentejo and Vicentine Coast Natural Park and the National Forest of the Maritime Dunes of Vila Real de Santo António [2, 6, 9]. This subspecies is an evergreen shrub, usually under 1 m tall, characterized by whorled leaves and marked morphological and reproductive plasticity [3, 4, 6, 10]. Although predominantly dioecious, hermaphroditic individuals occur in several populations, including El Asperillo, Vila Real de Santo António, and Praia de Mira [3, 4, 11]. Flowering takes place from February to April, and fruiting from June to September, with some latitudinal variation [3, 4, 11].

The edible fruits of *C. album*, traditionally consumed raw or transformed into juices, jams, or liqueurs, have been exploited since the early Neolithic, and ethnobotanical uses include antipyretic and anthelmintic properties [12, 13, 14]. Several studies have highlighted the high nutritional and phytochemical value of *C. album* berries, rich in polyphenols and other bioactive metabolites [8, 12, 13, 15, 16, 17, 18, 19, 20, 21, 22]. The consumption of these berries and derived products has been associated with antioxidant [13, 16, 17, 18, 20, 21, 22], antimicrobial [18], anticancer [20, 21], and neuroprotective effects [21, 22]. Leaves were traditionally used against scurvy [14], have a high phenolic content, and display relevant bioactivities, including antioxidant [17, 20, 22, 23, 24], antibacterial [24], cytoprotective [22], and cytotoxic effects [25, 26, 27, 28, 29]).

The survival of *C. album* is increasingly threatened by climate change, habitat degradation, coastal urbanization, tourism, invasive species, and potential overharvesting due to its ethnobotanical relevance [2, 11, 14, 30, 31, 32]. In Spain, particularly in Andalusia and Galicia, the species is already classified as vulnerable [5, 11], whereas in Portugal it remains unlisted as endangered [33]. The southernmost Portuguese populations are extremely localized and subjected to intense anthropogenic pressure in fragile coastal pine forests [9, 11], especially those from Vila Real de Santo António. Given these threats, understanding how environmental variation influences the phytochemical and bioactive profiles of distinct *C. album* ecotypes is essential to support conservation, reveal adaptive metabolic traits, and promote sustainable valorization of this endemic species. Investigating the leaves as an underexplored yet renewable source of bioactive compounds further provides insights into plant resilience under stress while fostering nature‐based applications that reduce exploitation.

In this context, the present study aimed to (1) evaluate and compare the phytochemical composition and bioactive properties of *C. album* leaves collected from two ecologically contrasting coastal habitats in southern Portugal, (2) determine how environmental variation influences metabolite profiles and associated biological activities, and (3) inform climate‐smart conservation and sustainable valorization by revealing the functional potential of leaves as a renewable, ecologically viable source of health‐promoting compounds.

## 2 Results and Discussion

### 2.1 Climatic Differences Between Collection Sites

*Corema album* is a perennial evergreen species whose physiological condition, mineral composition, and phytochemical profile may integrate environmental influences occurring over extended periods rather than reflecting only the meteorological conditions prevailing at the time of collection. Therefore, to provide a more ecologically meaningful characterization of the environmental conditions preceding sampling, the climatic assessment considered the period from January 2021 to January 2022. This 1 year period encompasses the seasonal cycle immediately preceding collection and provides information on the recent temperature, precipitation, solar radiation, and atmospheric humidity conditions potentially influencing plant growth, resource availability, and secondary metabolism. Nevertheless, because *C. album* is long‐lived, this period should be regarded as a characterization of the recent climatic conditions experienced by the studied populations rather than as a complete representation of the cumulative environmental conditions shaping their mineral and phytochemical profiles over multiple years.

The considered period (January 2021–January 2022) was characterized by atypical meteorological conditions in mainland Portugal within a broader European context. Although 2021 was not among the warmest years recorded in Europe, mean air temperature was above the long‐term average, while several regions of the Iberian Peninsula experienced persistent drier‐than‐average conditions. In Portugal, 2021 was classified as warm and dry, despite marked seasonal variability. Consecutive precipitation deficits in November and December contributed to the renewed expansion and intensification of meteorological drought. December was classified as very warm, and unusually high temperatures persisted from late December to early January 2022. Accordingly, January 2022, when the plant material was collected, was classified as warm and very dry [34, 35].

The climatic conditions recorded during the month of collection differed between the two sampling sites (Figure S1). CL1, located in Vila Real de Santo António, exhibited a higher minimum temperature than CL2, located in Aljezur (7.24°C vs. 4.55°C), as well as higher global solar radiation (9174 vs. 7750 kJ m^−^^2^). Precipitation was lower at CL1 than at CL2 during the same period (1.60 vs. 3.40 mm) [36]. Conversely, CL2 exhibited a higher maximum relative humidity than CL1 (97.7% vs. 88.9%), although its minimum relative humidity was slightly lower (45.0% vs. 49.3%). Maximum air temperatures were similar between the two locations.

These data indicate that, during the collection period, CL1 was comparatively warmer, drier, and more exposed to solar radiation, whereas CL2 experienced cooler conditions and greater atmospheric moisture availability. These short‐term observations are also broadly consistent with the contrasting climatic settings of the southeastern and western Algarve. However, the exceptionally dry conditions recorded in January 2022 limit the extent to which the collection‐month data can be considered representative of typical climatic conditions at either site. Accordingly, they should be interpreted together with annual or long‐term climatic patterns rather than used independently to establish climate‐driven relationships. These data describe the environmental conditions recorded during the monitoring period and should not be interpreted as long‐term climatic normals. Therefore, the observed phytochemical differences cannot be attributed exclusively to stable microclimatic differences between the two sites.

### 2.2 Mineral Composition

The most abundant minerals in *C. album* leaves were Ca (CL1 = 24.1 mg/g dw; CL2 = 11.1 mg/g dw) and K (CL1 = 3.86 mg/g dw; CL2 = 3.61 mg/g dw), followed by Mg (CL1 = 1.50 mg/g dw; CL2 = 2.00 mg/g dw) and Na (CL1 = 0.23 mg/g dw; CL2 = 1.33 mg/g dw). Among the trace elements, the highest concentrations were found for Mn (CL1 = 53.2 µg /g dw; CL2 = 170 µg/g dw) and Fe (CL1 = 100 µg/g dw; CL2 = 130 µg/g), Zn (CL1 = 10.7 µg/g dw; CL2 = 10.8 µg/g dw), and Cu (CL1 = 5.90 µg/g dw; CL2 = 3.77 µg/g dw) (Table 1). The contents of Ni and Cr were **≤** 2.35 µg/g dw, while Pb and Cd were below the limit of quantification (LOQ = 0.23 µg/g dw and LOQ = 0.09 µg/g dw, respectively; Table S1).

**TABLE 1:** Minerals content in leaf extracts of *C. album* ecotypes (CL1 and CL2).

Minerals		Ecotype	
		CL1	CL2
Macro elements (mg/g dw)	Ca	24.1 ± 0.12^a^	11.1 ± 0.20^b^
	K	3.86 ± 0.07^a^	3.61 ± 0.06^b^
	Mg	1.50 ± 0.04^b^	2.00 ± 0.04^a^
	Na	0.23 ± 0.06^b^	1.33 ± 0.02^a^
Trace elements (µg/g dw)	Mn	53.2 ± 1.02^b^	170 ± 4.49^a^
	Fe	100 ± 2.65^b^	130 ± 3.61^a^
	Zn	10.7 ± 1.10^a^	10.8 ± 0.78^a^
	Cu	5.90 ± 1.30^a^	3.77 ± 0.24^b^
	Ni	0.43 ± 0.11^b^	2.35 ± 1.14^a^
	Cr	0.45 ± 0.02^a^	0.48 ± 0.07^a^

Values represent the mean ± SD of at least three experiments (*n* = 3). Within the same row, values followed by different letters (a, b) are significantly different at *p* < 0.05 (Student's *t*‐test).

Mineral profiles differed significantly between ecotypes. CL1 showed higher Ca (over twice that of CL2) and K contents, whereas CL2 had greater levels of Mg and Na, the latter nearly six times higher. CL2 also accumulated more Mn, Ni, and slightly more Fe, while Cu was higher in CL1 and Zn and Cr remained similar between ecotypes.

Although both ecotypes were collected from coastal habitats in southern Portugal, differences in both microclimatic and edaphic conditions may contribute to the contrasting mineral profiles observed. CL2 (Aljezur) experiences cooler temperatures, higher humidity, and lower solar radiation, whereas CL1 (Vila Real de Santo António) is exposed to drier conditions and higher irradiance. However, mineral accumulation is also strongly influenced by soil physicochemical properties and nutrient availability. As soil composition was not analysed in the present study, the relative contribution of edaphic and microclimatic factors cannot be disentangled. Therefore, the observed mineral differences should be interpreted as potentially resulting from the combined influence of local environmental conditions and ecotype‐specific nutrient uptake and allocation. The pronounced Na enrichment in CL2 may reflect a salt‐tolerance strategy through vacuolar sequestration or osmotic use of Na^+^ while maintaining favourable Na^+^/K^+^ ratios [37, 38]. CL2 also contained more Mn, Fe, and Ni, micronutrients linked to antioxidant defence and metabolic activity [39], while Cu was higher in CL1 and Zn and Cr remained similar. These trends align with previous reports for *C. album* from distinct edaphoclimatic zones [40] and confirm that mineral allocation in *C. album* varies with organ and habitat, including soil properties, as also observed for berries [20, 41].

### 2.3 Extraction Yields and Phytochemical Content

#### 2.3.1 Extraction Yields and (Poly)Phenolic Content

The methanolic extracts of the two *C, album* ecotypes showed comparable extraction yields of 37.6% and 36.7% (w/w) for CL1 and CL2, respectively. However, comparable extraction yields reflect the total quantity of extracted material and do not necessarily indicate identical phytochemical compositions. In fact, clear differences in (poly)phenolic content were observed between the two ecotypes (Table 2). Ecotype CL1 displayed significantly higher levels of total phenolic content (TPC, 134 mg GAE/g dw), total flavonoids content (TFC, 116 mg QE/g dw), hydroxycinnamic acid (HA, 144 mg CAE/g dw), and condensed tannins content (CTC, 79.6 mg CE/g dw) than CL2 (TPC: 105 mg GAE/g dw; TFC: 95.7 mg QE/g dw; HA: 120 mg CAE/g dw; CTC: 69.1 mg CE/g dw). These differences are likely associated with the contrasting climatic conditions at the collection sites (Table 1). CL1 is exposed to stronger environmental stress, as “Vila Real de Santo António” experienced higher solar radiation and lower precipitation during the sampling period. Such abiotic pressures are known to enhance the biosynthesis of polyphenols as protective metabolites involved in drought tolerance, photoprotection, and mitigation of photooxidative stress [23, 42, 43, 44]. Flavone and flavonol (F/F) contents were similar between ecotypes (CL1: 87.4 mg QE/g dw; CL2: 83.8 mg QE/g dw), suggesting that these compounds may be less responsive to microclimatic variation or are more tightly regulated by developmental or genetic factors [42, 45].

**TABLE 2:** (Poly)phenolic content in leaf extracts of *C. album* ecotypes (CL1 and CL2).

Group of (poly)phenolics	Ecotypes	
	CL1	CL2
TPC	134 ± 4.17**^a^**	105 ± 8.51**^b^**
TFC	116 ± 8.82**^a^**	95.7 ± 5.38**^b^**
F/F	87.4 ± 7.16**^a^**	83.8 ± 8.91**^a^**
HA	144 ± 8.50**^a^**	120 ± 7.68**^b^**
CTC	79.6 ± 2.60**^a^**	69.1 ± 5.97**^b^**

TPC: total phenolics content, mg GAE/g dw (GAE: gallic acid equivalents); TFC: total flavonoids content, mg QE/g dw (QE: quercetin equivalents); F/F: flavones/flavonols, mg QE/g dw; HA: hydroxycinnamic acids, mg CAE/g dw (CAE: caffeic acid equivalents); CTC: condensed tannins content, mg CE/g dw (CE: catechin). Values represent the mean ± SD of at least three experiments performed in triplicate (*n* = 9). Within the same row, values followed by different letters (a, b, c) are significantly different at *p*< 0.05 (Student's t‐test).

Overall, these results highlight the strong influence of local environmental conditions, particularly solar irradiance and water availability, on the phenolic profile of *C. album*. This pattern aligns with observations in other Ericaceae species, such as *Arbutus unedo*, where substantial intra‐ and inter‐population variation in phenolic content has been attributed to eco‐climatic heterogeneity [46, 47].

#### 2.3.2 Metabolomic Profile

Untargeted metabolomic analysis revealed the presence of 49 putatively annotated compounds in the methanolic extracts of *C. album* leaves (Table S2), mainly (poly)phenols (73.5% of the total). Flavonoids were the most abundant subclass (55.1% of the total compounds), consistent with previous reports for this species [17, 22, 23, 29]. Among these, flavonols were the dominant subgroup, followed by chalcones, flavones and flavanones, and anthocyanins. Additional subclasses of (poly)phenolics included phenolic acids (hydroxybenzoic and hydroxycinnamic acids) and single representatives of a phenolic glycoside, a coumarin, and an anthraquinone. Non‐phenolic compounds were also putatively identified, namely organic and fatty acids, one sugar, and one amide, which are mainly involved in primary metabolism, osmoprotection, and structural stabilization of leaf tissues.

**FIGURE 1:**
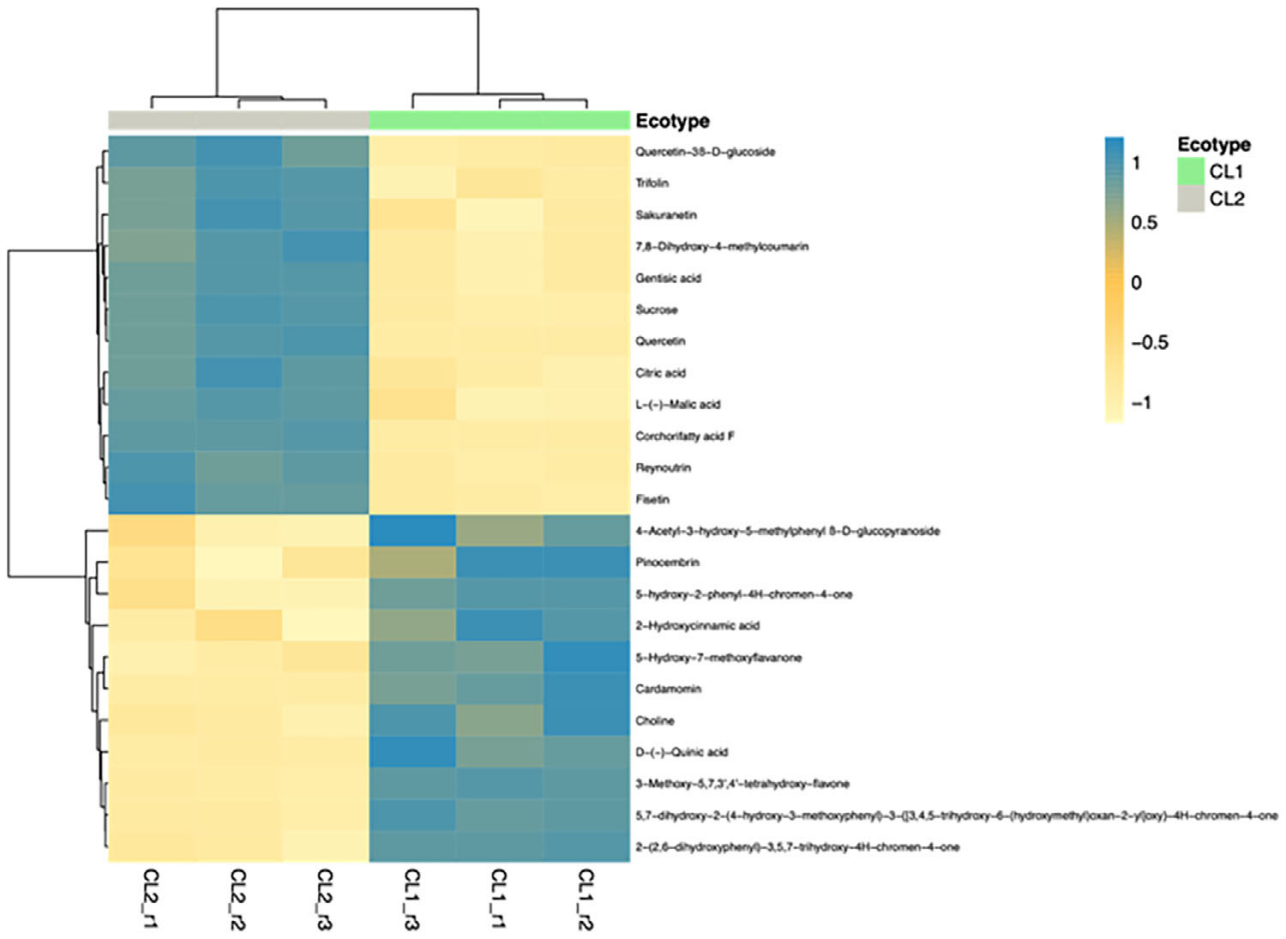
Hierarchical clustering analysis of annotated features. Colors from yellow to blue indicate the normalized relative abundance values of metabolites from low to high according to the scale bar. Ecotypes, CL1 (green) and CL2 (gray), are arranged in columns.

Despite the similar metabolite profiles observed in both ecotypes, the relative abundance of individual metabolites varied significantly, suggesting the influence of environmental factors on secondary metabolism [42, 43, 48]. CL1 exhibited higher normalized relative abundance of (poly)phenols, including several structurally diverse flavonoids such as pinocembrin, cardamomin, 5‐hydroxy‐7‐methoxyflavanone, and various hydroxylated flavonols and flavones (Figure 1). The compounds 2‐hydroxycinnamic acid (phenolic acid) and 4‐4‐acetyl‐3‐hydroxy‐5‐methylphenyl β‐D‐glucopyranoside (phenolic glycoside) were also more abundant in CL1. In contrast, flavonols, especially quercetin and its glycoside, fisetin, reynoutrin, and trifolin, were more abundant in CL2, as well as sakuranetin (flavanone) and gentisic acid (hydroxybenzoic acid) (Figure 1). These findings are consistent with the (poly)phenolic profiling, in which CL1 showed higher TPC, TFC, and HA values compared to CL2 (Table 2). CL2 also showed higher abundance levels of sucrose (carbohydrate), citric acid and L‐(‐)‐malic acid (organic acids), 7,8‐dihydroxy‐4‐methylcoumarin (coumarin), and corchorifatty acid (fatty acid), whereas CL1 had higher relative abundances in choline (amine) and D‐(‐)‐quinic acid (organic acid) (Figure 1). Although condensed tannins were not directly tentatively identified in the metabolomic dataset, several flavonoids and glycosylated derivatives, including naringenin chalcone, sakuranetin, pinocembrin, and quercetin or myricetin conjugates, as well as other structurally related phenolics, may account for the positive responses observed in the colorimetric assays, which are less specific and respond to a broader spectrum of phenolic structures. These comparisons are based on relative signal abundance and should not be interpreted as absolute differences in compound concentration.

The predominance of flavonols and flavones in the metabolomic profile supports previous studies in *C*. *album* leaves [17, 22, 23, 29], which reported the presence of quercetin, myricetin, and kaempferol and their derivatives, along with phenolic acids. By contrast, proanthocyanidins, (epi)catechin, lignans, and stilbene derivatives were not detected in the present study, possibly due to differences in extraction methods, harvest time, or detection thresholds.

**FIGURE 2:**
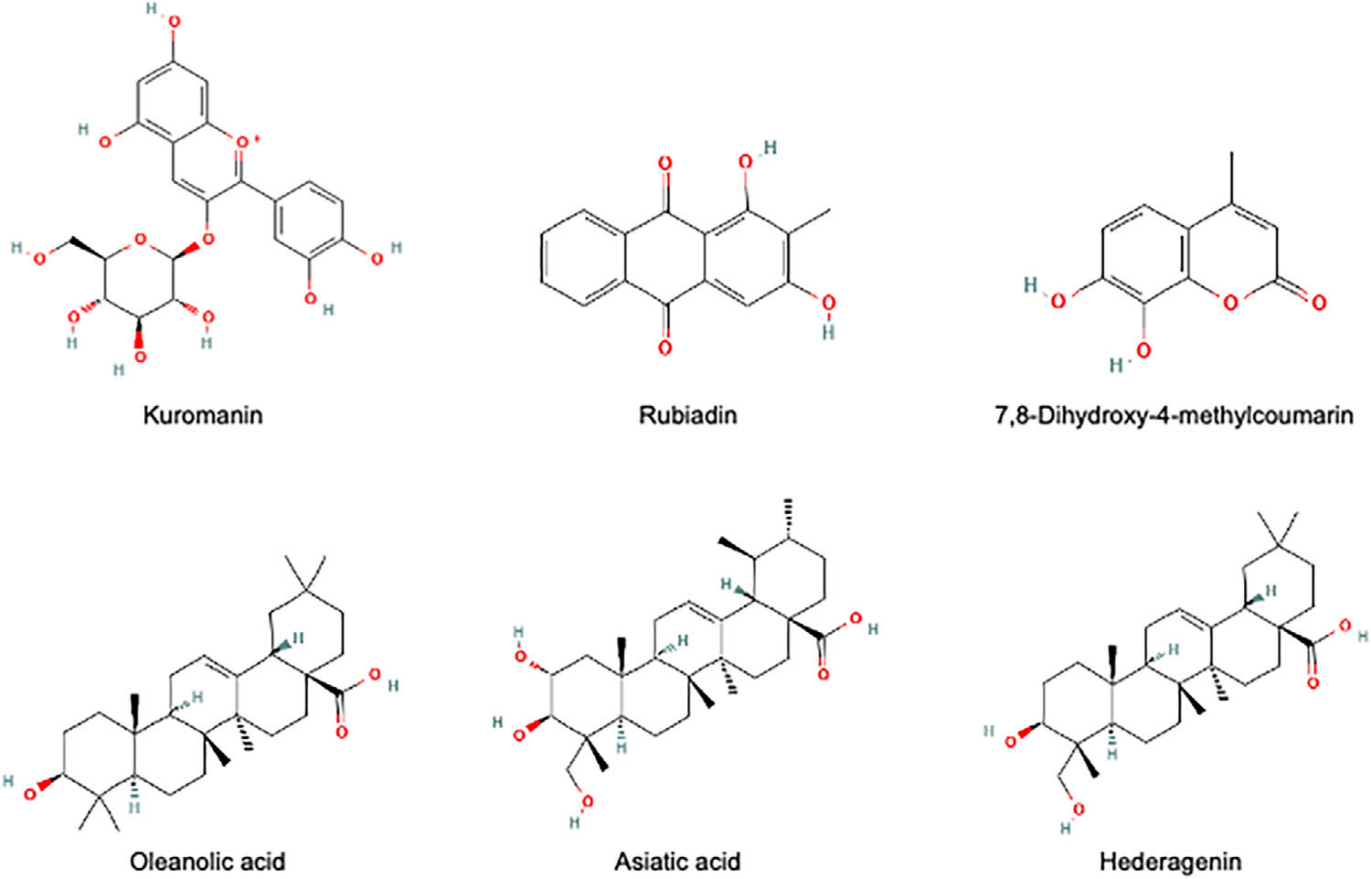
Chemical structures of secondary metabolites newly identified in the leaves of *C. album*, including anthocyanins (kuromanin), an anthraquinone (rubiadin), a coumarin (7,8‐dihydroxy‐4‐methylcoumarin), and pentacyclic triterpenoids (oleanolic acid, asiatic acid, and hederagenin). Structures were retrieved from PubChem [49].

Results also revealed the presence of anthocyanins, anthraquinones, coumarins, and triterpenes in the leaves of *C. album*, including pentacyclic triterpenoids of the oleanane and ursane types, such as oleanolic and asiatic acids, or structurally related analogues [49], which had not been previously reported in the leaf tissues of this species (Figure 2). These classes of compounds, except for anthraquinones, which have been reported in various organs of the *Ericaceae* family, namely *A. unedo* [50, 51, 52], *Rhododendron arboreum* [53], and *Erica arborea* [54], were previously identified in the berries of *C*. *album* [8, 12, 13, 18, 19, 21], and are recognized for their roles in plant defence and photoprotection, as well as for their pharmacological potential, including antioxidant, anti‐inflammatory, and anticancer activities [8, 55, 56, 57, 58, 59, 60]. The tentative annotation of 18‐β‐glycyrrhetinic acid (18β‐GA) should be interpreted with caution, as it shares a fragmentation pattern with other oleanane‐type triterpenes. Given the absence of prior reports of 18β‐GA in the Ericaceae family, this signal may correspond to a structurally related isomer rather than the compound itself.

Overall, the marked differences in metabolite abundance between CL1 and CL2 reinforce the strong influence of local edaphoclimatic conditions on the secondary metabolism of *C. album*. These findings align with observations in related *Ericaceae* species, such as *A*. *unedo* [46, 47, 61], where metabolomic variability has likewise been linked to environmental and geographic gradients. Taken together, these results highlight the ecological and functional significance of chemical diversity in wild plant populations and underscore the importance of integrative approaches, combining metabolomics, environmental characterization, and phenological data, to fully understand and valorize their adaptive potential.

Seasonal and phenological effects should therefore be considered when interpreting the present metabolomic profiles, as flowering and fruiting may alter carbon allocation and secondary metabolite accumulation in leaves. Moreover, although no obvious morphological differences were observed between leaves from the two ecotypes, detailed morphometric and anatomical analyses were not conducted. Therefore, subtle structural differences potentially associated with the observed phytochemical variability cannot be excluded. In addition, future studies should identify and tag individuals during flowering and subsequently compare male, female, and hermaphroditic plants, if appropriate, to determine whether sexual type may contribute to the observed phytochemical variability.

### 2.4 Antioxidant Activity

The antioxidant activity of *C. album* extracts was determined through a set of in vitro assays reflecting distinct mechanisms of action, including radical scavenging (RSA in ABTS•^+^ and DPPH•), redox potential (FRAP), and metal chelation (CCA and ICA) (Table 3). The extracts exhibited strong RSA, with no statistically significant differences between ecotypes, with EC_50_ values of 0.10 and 0.11 mg/mL for 2,2′‐azino‐bis(3‐ethylben zothiazoline‐6‐sulfonic acid (ABTS•^+^) and 0.17 and 0.16 mg/mL for 2,2‐diphenyl‐1 picrylhydrazyl (DPPH•, CL1 and CL2, respectively). The ferric reducing antioxidant power (FRAP) was also comparable between ecotypes, with EC_50_ of 0.08 for CL1 and 0.09 mg/mL for CL2. By contrast, significant differences were observed in the CCA, with CL1 displaying higher chelating capacity (EC_50_  =  2.21 mg/mL) than CL2 (EC_50_  =  2.64 mg/mL). However, neither extract reached 50% of ICA at the maximum concentration tested (10 mg/mL). The reduced Fe^2^^+^ chelation contrasts with the strong radical scavenging and reducing power, suggesting that the antioxidant mechanisms in *C. album* leaves are primarily driven by redox‐active compounds rather than direct metal complexation.

**TABLE 3:** In vitro antioxidant activities of methanolic leaf extracts of *C. album* ecotypes (CL1 and CL2). Results are expressed as EC_50_ values (mg/mL).

Assay	Ecotypes		*Positive control
	CL1	CL2	
RSA‐ABTS•^+^	0.10 ± 0.03**^b^**	0.11 ± 0.01**^b^**	0.02 ± 0.00**^a^**
RSA‐DPPH•	0.17 ± 0.02**^b^**	0.16 ± 0.02**^b^**	0.03 ± 0.00**^a^**
CCA	2.21 ± 0.18**^b^**	2.64 ± 0.19**^c^**	0.12 ± 0.01**^a^**
ICA	—	—	0.01 ± 0.00
FRAP	0.08 ± 0.00**^a^**	0.09 ± 0.01**^a^**	n.a.

“‐”: indicates activity lower than 50% at 10 mg/mL. “n.a.”—not applicable *Positive controls: RSA on ABTS•^+^ and DPPH•—Gallic acid (GAE); CCA and ICA—ethylenediaminetetraacetic acid (EDTA); FRAP—butylated hydroxytoluene (BHT, E320). Values represent the mean ± SD of at least three experiments performed in triplicate (*n* = 9). Within the same row, values followed by different letters (a, b, c) are significantly different at *p* < 0.05.

The strong RSA and FRAP activities observed are in line with the (poly)phenolic composition of both extracts, particularly the high abundance of flavonoids bearing catechol‐type structures in the B‐ring, such as quercetin, myricetin, and fisetin, and their glycosides (e.g., reynoutrin, 3‐O‐β‐D‐galactopyranoside e quercetin‐3β‐D‐glucoside). These substitution patterns are known to enhance antioxidant performance by stabilizing unpaired electrons and promoting hydrogen or electron donation [46, 62].

The comparable levels of flavonols and flavones in CL1 and CL2 likely explain the absence of significant differences in RSA and FRAP. In contrast, the higher HA, CTC, and TPC values observed in CL1 may contribute to its enhanced CCA activity, as these phenolic classes contain multiple hydroxyl groups and aromatic rings that enable moderate metal‐chelating capacity, particularly toward transition metals such as Cu^2^^+^ [63, 64]. The metabolomic profile further showed a higher relative abundance of several flavonoids in CL1, including cardamomin, pinocembrin, 5‐hydroxy‐7‐methoxyflavanone, and other hydroxylated flavones and flavonols. Among these, hydroxylated flavonoids are the most plausible contributors to Cu^2^^+^ chelation, as hydroxyl and carbonyl groups can provide coordination sites for metal binding. However, because the chelating activity was evaluated using crude extracts, the contribution of individual metabolites cannot be established, and synergistic interactions among multiple phenolic compounds may also be involved. On the other hand, the reduced Fe^2^^+^ chelation capacity observed in both extracts can be attributed to the prevalence of flavonoids in glycosylated or methoxylated forms (e.g., 5‐hydroxy‐7‐methoxyflavanone), which reduce the number of available coordination sites essential for Fe^2^^+^ binding [63, 64].

Comparative literature supports the antioxidant capacity of *C. album* leaves. Ferreira [65] reported slightly lower EC_50_ values in methanolic extracts of fresh leaves (0.08–0.09 mg/mL DPPH), suggesting stronger RSA than those in this study. Comparisons with other Ericaceae species further contextualize the results. In *A. unedo*, EC_50_ values for ABTS•^+^ and DPPH• range from 0.004 to 0.95 mg/mL depending on genotype, extraction method, and site conditions [46, 47, 61, 66]. These findings reinforce the influence of environmental conditions and phytochemical composition on antioxidant performance in *C. album*. Overall, the antioxidant potential of the leaves appears to be driven mainly by reducing power and free‐radical scavenging, both closely associated with their phenolic and flavonoid profiles. Metal chelation, particularly of Fe^2^^+^, plays a more limited role, likely constrained by structural features of the predominant compounds.

### 2.5 Principal Component Analysis

**FIGURE 3:**
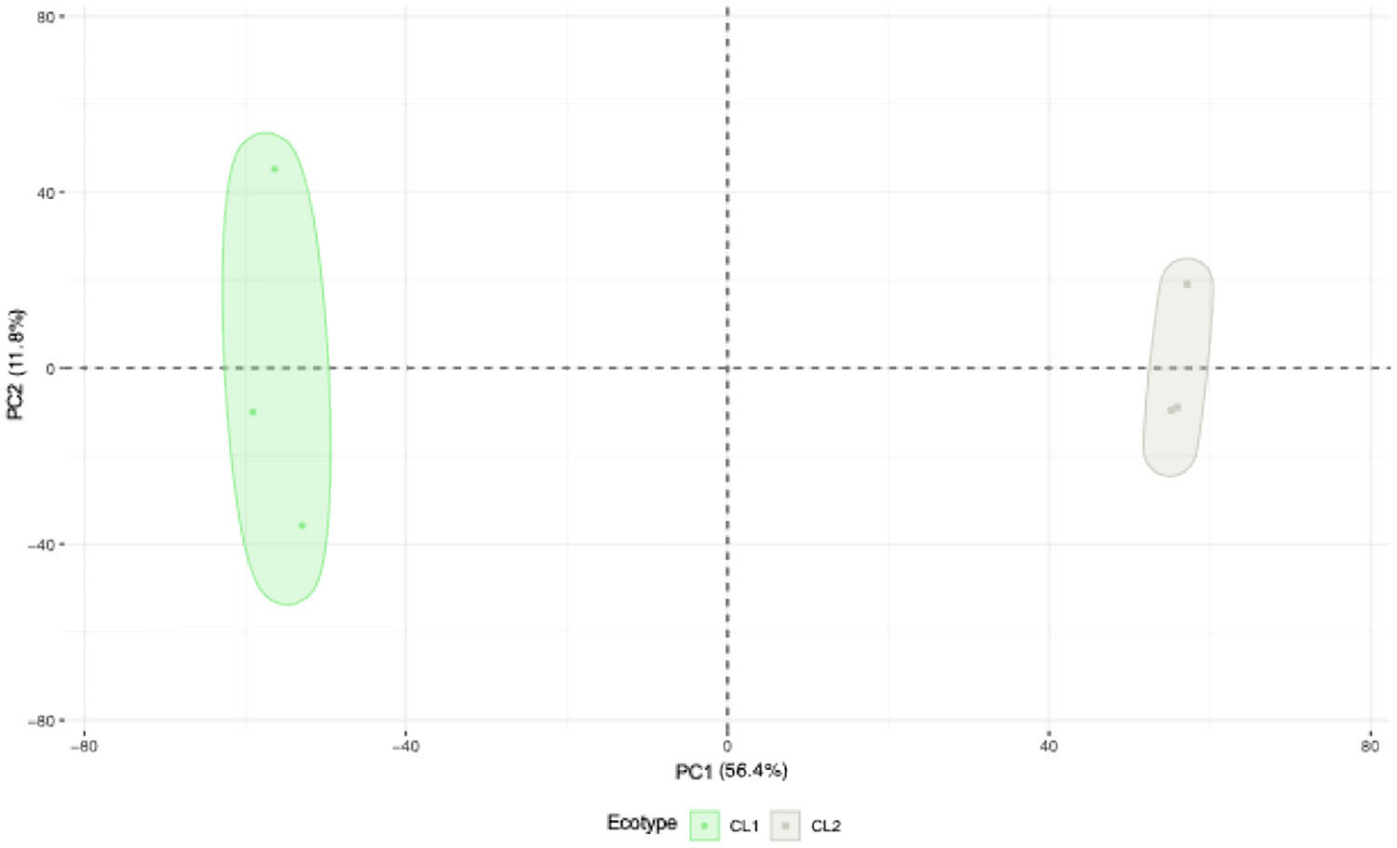
PCA showing the dissimilarities between ecotypes' metabolomes. The *x*‐ and *y*‐axes represent principal components 1 and 2, respectively, in brackets are the percentages of the overall variance explained by each principal component.

**FIGURE 4:**
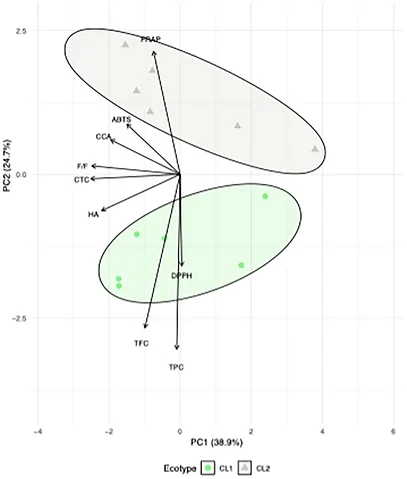
PCA of the (poly)phenolic contents (TPC, TFC, F/F, HA, and CTC) and of the antioxidant activity (ABTS•^+^, DPPH•, CCA, and FRAP) of the extracts from two *C*. *album* ecotypes (CL1 and CL2) collected from different locations (“Vila Real de Santo António” and “Aljezur”).

To visualize the differences in the phytochemical composition and the biological activities *C. album* leaves from the two ecotypes, two principal component analysis (PCA) biplots were constructed. The first PCA was based on the full metabolomic profiles of CL1 and CL2 (Figure 3), while the second incorporated (poly)phenolic contents (TPC, TFC, F/F, HA, and CTC) and antioxidant activities (ABTS•^+^, DPPH•, FRAP, and CCA) (Figure 4).

The PCA of metabolomic data (Figure 3) explained 68.2% of the observed variance (PC1: 56.4%; PC2: 11.8%) and revealed a clear separation between the two ecotypes, indicating distinct chemical profiles at the metabolome level. Similarly, the PCA score plot of the (poly)phenolic contents and antioxidant parameters explained 63.6% of the variance (PC1: 38.9%; PC2: 24.7%) and revealed a clear distinction between CL1 and CL2 (Figure 4). CL1 was positioned in close association with TPC, TFC and DPPH• scavenging activity, reflecting its overall higher (poly)phenolic content and radical scavenging capacity and a trend toward stronger radical scavenging capacity. In contrast, CL2 appeared more closely related to FRAP, indicating a slightly higher ferric reducing capacity and overall redox‐based antioxidant potential. CTC, CCA, F/F, HA, and ABTS•^+^ displayed an intermediate loading, not strongly contributing to the discrimination between ecotypes. CL1 samples formed a tighter cluster, suggesting greater internal consistency, whereas CL2 samples showed higher dispersion, indicating greater variability among the analysed samples. This pattern may reflect natural biological heterogeneity within the CL2 population and/or greater spatial variability in local environmental conditions. However, given the limited number of individuals and environmental variables assessed, the underlying drivers of this dispersion cannot be conclusively determined. Future studies including larger population‐level sampling, repeated collections across seasons, and detailed soil and microenvironmental characterization will be necessary to determine whether this variability represents stable intra‐population heterogeneity or environmentally driven temporal and spatial variation.

### 2.6 Cytotoxicity Activity

The extracts showed cytotoxic activity in all tested cell lines, with clear differences between ecotypes (Table 4). CL1 exhibited higher cytotoxicity than CL2, as reflected by its lower EC_50_ values across the panel. These differences are consistent with the distinct phytochemical signatures of the ecotypes, particularly the higher abundance of (poly)phenolic compounds in CL1, including hydroxycinnamic acids and diverse flavonoids. Compounds such as cardamomin and pinocembrin, which were more abundant in CL1, have been reported to affect mitochondrial function, redox balance, and DNA integrity in tumor cells, and may therefore contribute to the observed cytotoxicity [13, 25, 26, 27, 67, 68, 69]. However, because cytotoxic effects were also observed in the nontumoral HEK293 cell line, the present results should be interpreted as evidence of general cytotoxic activity rather than selective anticancer effects. Further work using bio‐guided fractionation and mechanistic assays (e.g., apoptosis/necrosis discrimination, mitochondrial function, and ROS generation), together with selectivity assessment in additional tumor and nontumor cell models, will be essential to identify the most active metabolites and clarify their modes of action.

**TABLE 4:** EC_50_ values (µg/mL) of leaf extracts from *C. album* ecotypes (CL1 and CL2) in HEK 293, B16 4A5, HepG2 and SH‐SY5Y cell lines.

Cell lines	Ecotypes	
	CL1	CL2
HEK 293	32.2 ± 3.49^a^	79.4 ± 8.15^b^
B16 4A5	90.4 ± 4.81	—
HepG2	58.6 ± 7.29^a^	104 ± 8.52^b^
SH‐SY5Y	82.4 ± 3.64	—

Values represent the mean ± SD of at least three experiments performed in triplicate (*n* = 9). Lower case letters indicate significant differences between ecotypes within the same cell line (*p*< 0.05).

From an applied perspective, the pronounced differences in cytotoxic potency highlight the distinct biological profiles of the two ecotypes and may support differentiated valorization strategies. CL1 showed a stronger cytotoxic profile, whereas CL2 exhibited lower cytotoxicity and a profile more closely associated with antioxidant activity. Nevertheless, the lack of demonstrated selectivity toward tumor cells precludes conclusions regarding anticancer potential at this stage. These findings underscore the importance of intraspecific chemical variability, not only from an ecological perspective but also for guiding the future investigation and potential application of *C. album* bioresources.

## 3 Conclusions

This study provides new insights into the variation in leaf composition and bioactivity between two *C. album* subsp. *album* ecotypes from contrasting coastal environments in southern Portugal. Despite being taxonomically identical, CL1 and CL2 differed markedly in their mineral composition, (poly)phenolic profiles, metabolomic signatures, and biological activities. CL1, collected from the drier and more irradiated site, had higher contents of total (poly)phenolics, flavonoids, hydroxycinnamic acids, and condensed tannins, as well as a greater relative abundance of several flavonoids and phenolic acids. These characteristics were accompanied by stronger copper‐chelating activity and greater cytotoxicity. In contrast, CL2, collected from the cooler and more humid site, contained higher levels of Mg, Na, Mn, and Fe, displayed a distinct metabolic profile, and showed lower cytotoxicity. Both ecotypes exhibited strong radical‐scavenging and ferric‐reducing activities, possibly related to their shared abundance of flavonols and flavones containing catechol‐type structures. Fe^2^^+^ chelation was limited, potentially due to the glycosylation or methoxylation of key phenolic compounds. Overall, the differences observed between the two ecotypes may result from the combined influence of local environmental conditions, including microclimatic and edaphic factors, together with population‐specific responses. The results highlight the potential of *C. album* leaves as a renewable source of bioactive compounds and the importance of considering ecotype‐specific profiles in conservation, valorization, and bioprospecting strategies. However, the effects of leaf harvesting on plant recovery and reproductive fitness remain unknown. Harvesting from wild populations, particularly in protected dune habitats, should therefore not be considered without prior ecological assessment. Cultivation may provide a more sustainable source of leaves and fruits while reducing pressure on natural populations and could also support propagation and carefully planned population reinforcement programmes.

Finally, although CL1 showed greater cytotoxicity than CL2, both extracts reduced the viability of nontumoral HEK293 cells. Therefore, no conclusions regarding selective anticancer activity can be drawn at this stage. Further studies should assess selectivity toward tumor cells, identify the compounds responsible for the observed activity, and clarify their mechanisms of action in relevant biological models.

## 4 Experimental Section

### 4.1 Plant Material

**FIGURE 5:**
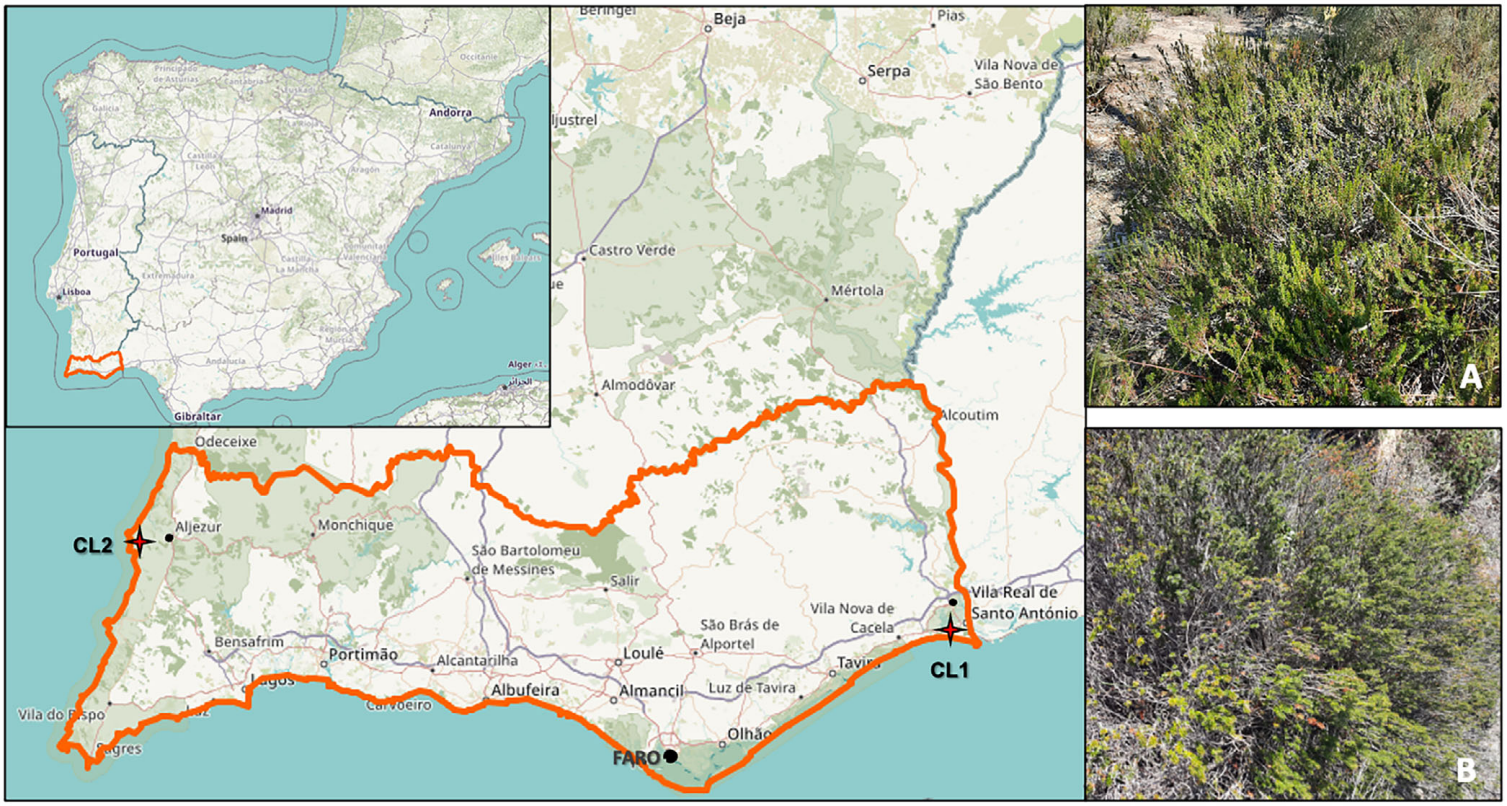
Geographical location of *C. album* subsp. *album* ecotype harvesting sites in the Algarve region, southern Portugal. (A) CL1 – “National Forest of the Maritime Dunes of Vila Real de Santo António,” Vila Real de Santo António. (B) CL2 – “Southwest Alentejo and Vicentine Coast Natural Park,” Aljezur [70].Map data OpenStreetMap contributors, licensed under the Open Data Commons Open Database License (ODbL) 1.0.

Leaves from *C. album* subsp. *album* were hand‐collected in winter (January 2022) from at least seven adult plants in populations located in two areas of southern Portugal (Algarve): ecotype CL1, in Vila Real de Santo António, within the National Forest of the Maritime Dunes of Vila Real de Santo António on the eastern coast (37°10'58.71“N; 7°25'21.09”W); and ecotype CL2, in Aljezur, within the Southwest Alentejo and Vicentine Coast Natural Park on the southwestern coast (37°17'1.54“N; 8°51'37.89”W) (Figure 5) [70]. Once in the laboratory, leaves were washed with tap water, freeze‐dried, powdered, and stored at −20°C until analysis. The plant material was taxonomically identified as *C. album*  subsp. *album* by Dr. Luísa Custódio (CCMAR). Voucher specimens representing the two ecotypes were deposited in the XtremeBio Lab Herbarium (CCMAR, University of Algarve, Faro, Portugal) under the voucher codes XBH44.1 (CL1) and XBH44.2 (CL2). No obvious morphological differences were observed between the leaves of the two ecotypes at the time of collection. However, detailed morphometric or anatomical analyses were not performed. January was selected to standardize sampling during the winter vegetative period, before the onset of flowering and fruit development, thereby minimizing potential phenological effects on leaf metabolism. As seasonal changes associated with reproductive development may influence resource allocation and secondary metabolism, leaf phytochemical profiles during flowering and fruiting may differ from those observed in the present study. Because leaf sampling was conducted in January, before the main flowering period of *C. album*, the sampled individuals could not be reliably classified according to sexual type based on reproductive morphology. Therefore, the possible occurrence of hermaphroditic individuals within the sampled populations could not be determined, and sex‐related differences in phytochemical profiles were not assessed.

### 4.2 Characterization of the Collection Sites

#### 4.2.1 Vila Real de Santo António

Samples from CL1 were collected in the National Forest of the Maritime Dunes of Vila Real de Santo António (southern Portugal). This is a coastal protected area with a mild Mediterranean climate, hot dry summers (18°C–30°C), mild winters (9°C–16°C), irregular rainfall mainly in autumn–spring, and high annual sunshine (≈ 3000–4000 h) [71, 72]. The forest is dominated by *Pinus pinaster*, with scattered native species such as *Ceratonia siliqua*, *Quercus suber*, *Pinus pinea*, and *Quercus rotundifolia*. Dune habitats host characteristic vegetation, including *Otanthus maritimus*, *Eryngium maritimum*, *Cakile maritima*, *Ammophila arenaria*, and *Thymus carnosus* [71].

#### 4.2.2 Aljezur

Samples from ecotype CL2 were collected in Aljezur, within the Southwest Alentejo and Vicentine Coast Natural Park, a coastal protected area in southern Portugal characterized by Mediterranean climate conditions with strong Atlantic influence. The region presents mild winters (9°C–17°C), cool summers (18°C–26 °C), low annual precipitation (≈ 400–500 mm), and high solar exposure (≈ 2800–3000 h/year) [72, 73]. The local microclimate is shaped by seasonal north‐westerly winds and coastal fogs. Vegetation includes native cork and Portuguese oaks (*Quercus suber* and *Q. faginea*), strawberry tree (*A. unedo*) and endemic dune flora, including *Biscutella vicentina*, *Centaurea vicentina*, *Cistus palhinhae*, and *Diplotaxis vicentina*, alongside introduced species such as *P. pinaster* and *Eucalyptus globulus* [73].

### 4.3 Mineral Composition

Samples (250 mg) of freeze‐dried biomass were mixed with nitric acid (6 mL), digested on a Microwave Digestion System (CEM SP‐D80) for 10 min at 180°C, diluted (1:10) with ultra‐pure water, and analysed by atomic emission spectroscopy using a Microwave Plasma‐Atomic Emission Spectrometer (MP‐AES; Agilent 4200 MP‐AES, Agilent Victoria, Australia) [74]. Standards were prepared from certified standard solutions of the elements calcium (Ca), magnesium (Mg), potassium (K), sodium (Na), cadmium (Cd), chromium (Cr), copper (Cu), iron (Fe), lead (Pb), manganese (Mn), nickel (Ni), and zinc (Zn). The calibration ranges were 0.25–10 mg/L for Ca, K, Fe, Mg, and Na, and 25–1000 µg/L for Cd, Cr, Cu, Pb, Mn, Ni, and Zn.

### 4.4 Preparation of the Extracts

Dried ground samples were extracted overnight under stirring with methanol (1:40 w/v), in the dark at room temperature (RT). The extracts were filtered using Whatman No. 4 filter paper, and the methanol was removed in a rotavapor under reduced pressure at 35°C. For metabolomic profile dried extracts were weighed, dissolved in pure methanol at a concentration of 2 mg/mL, and filtered through 0.22 µm PTFE syringe filters before injection. For the remaining analysis, dried extracts were dissolved in methanol at 10 mg/mL and stored at 4°C. The extraction yield was calculated as the mass of dried extract relative to the initial mass of dried plant material and expressed as a percentage.

### 4.5 Phytochemical Analysis

#### 4.5.1 Determination of TPC

TPC was determined by the Folin–Ciocalteu (F–C) method, as previously described by Rodrigues et al. [75]. In brief, the extracts (5 µL; 10 mg/mL) were mixed with F–C reagent (100 µL; 1:10 in distilled water, v/v) and incubated for 5 min at RT. Then, 100 µL of sodium carbonate (75 g/L, w/v) was added, and after incubation for 90 min at RT the absorbance was measured at 725 nm on a microplate reader (Biochrom EZ Read 400, Santa Clara, CA, USA). Gallic acid was used as the standard for the calibration curve at concentrations ranging from 0.001 to 1 mg/mL (*r* ^2^ = 0.998), and TPC was expressed as milligrams of gallic acid equivalents per gram of dry weight (mg GAE/g, dw).

#### 4.5.2 Determination of TFC

TFC was estimated by the aluminum chloride (AlCl_3_) assay adapted to 96‐well microplates, as described in Rodrigues et al. [75]. Briefly, the extracts (50 µL; 10 mg/mL) of extracts were mixed with 50 µL of aluminium chloride (2%), incubated for 10 min at RT and the absorbance measured at 420 nm on a microplate reader (Biochrom EZ Read 400, Santa Clara, CA, USA). Quercetin was used as standard for the calibration curve, at concentrations between 0.002 and 0.5 mg/mL (*r* ^2^ = 0.999). Results were expressed as milligrams of quercetin equivalents per gram of dry weight (mg QE/g dw).

#### 4.5.3 Determination of F/F Content

F/F was quantified according to a previously described method by Rodrigues et al. [75]. Fifty microliters of the extracts (10 mg/mL) were mixed with 50 µL of 2% AlCl_3_–ethanol solution and incubated for 1 h at RT. The absorbance was measured at 420 nm on a microplate reader (Biochrom EZ Read 400, Santa Clara, CA, USA). A calibration curve was produced with quercetin at concentrations ranging between 0.01 and 1 mg/mL (*r* ^2^ = 0.9969). Results were expressed as milligrams of quercetin equivalents per gram of dry weight (mg QE/g, dw).

#### 4.5.4 Determination of HA

HA were estimated as described by Rodrigues et al. [75], The extracts (20 µL; 10 mg/mL) were mixed with aqueous ethanol (20 µL; 95%, v/v) containing hydrochloric acid (0.1%, v/v), and hydrochloric acid (160 µl; 2%, v/v). The absorbances were measured at 320 nm on a microplate reader (Biochrom EZ Read 400, Santa Clara, CA, USA). The calibration curves were prepared using different concentrations of caffeic acid (0.002–0.5 mg/mL; *r* ^2^ = 0.999). Results were expressed as caffeic acid equivalents per gram of dry weight (mg CAE/g dw).

#### 4.5.5 Determination of CTC

CTC was evaluated by the 4‐dimethylaminocinnamaldehyde–hydrochloric acid (DMACA–HCl) method, according to Rodrigues et al. [75]. Briefly, 10 µl of the extracts (10 mg/mL) were mixed with 200 µl of DMACA (1% in methanol, w/v) and 100 µl of hydrochloric acid (37%, v/v). After an incubation of 15 min at RT, absorbance was measured at 640 nm on a microplate reader (Biochrom EZ Read 400, Santa Clara, CA, USA). A calibration curve was prepared using catechin as a standard with concentrations ranging 0.004–1 mg/mL (*r* ^2^ = 0.997). Results were expressed as milligrams of catechin equivalents per gram of dry weight (mg CE/g dw).

#### 4.5.6 Metabolomic Profile

The chemical profiles of the extracts were performed on a Thermo Scientific UltiMate 3000 UHPLC, coupled to an Orbitrap Elite (Thermo Fisher Scientific, Waltham, MA, USA) mass spectrometer with a Heated Electrospray Ionization source (HESI‐II; Thermo Scientific). Briefly, extracts (5 µL) were injected onto a Thermo Scientific Accucore RP‐18 column (2.1 × 100 mm, 2.6 µm) and separated in a 40 min run with a flow of 0.30 mL/min. The mobile phase consisted of ultra‐pure LC‐MS grade water (A) and LC‐MS grade acetonitrile (B), both containing 0.1% formic acid. The gradient (v/v %) started with 100% of A for 2 min, and the B/A ratio progressively increased linearly to 30% B in 13 min, then to 100% B in 16 min. Finally, 100% B was maintained for 4 min. The mobile phase then returned to 100% of A in 1 min, and the column was stabilized at 100% of A for 4 min before the next run. Data were acquired under positive and negative ionization modes (HESI^+^/^−^) using the parameters reported in Silva et al. [76], and processed in Xcalibur v4.1 Qual Browser (Thermo Scientific). A solvent control, consisting of methanol 100 v/v %, and a quality control, consisting of a mixture of equal‐volume aliquots from all *C. album* extracts, were run.

Thermo RAW data were analysed separately with Compound Discoverer 3.2 (Thermo Fisher Scientific) using the untargeted workflow named “Untargeted Metabolomics with statistics detect unknowns with ID using Online Database and mzLogic” to perform retention time alignment and unknown compound identification using publicly available databases. The “Detect Unknown Compounds” node was used with default parameters, with the exception of mass tolerance (10 ppm) and minimum peak intensity (1000). The “Search ChemSpider” node was added by using Natural Products Atlas [77], Lipid Maps [78], KEGG [79], DrugBank [80], Carotenoids Database [81], Human Metabolome Database [82], Phenol Explorer [83], and BioCyc [84] online databases. The solvent control was used for background subtraction and noise removal. The resulting “cleaned‐up” feature list was normalized to the median signal intensity of each sample before statistical analyses. At first, an ANOVA test was carried out in Compound Discoverer to evaluate statistical differences (*p* < 0.05) among the metabolomes of *C. album* ecotypes. Bonferroni correction for *p* value and fold‐change of 1 were used for ANOVA. A PCA and a PERMANOVA test (*n* permutations = 999) were performed based on Euclidean distances to examine sample clustering using “vegan” and “ggplot2” packages in R‐Statistics 3.5.3. Feature annotation followed the convention reported in Sumner et al. [85]. However, in this work we relied on a conservative approach for final feature annotation. Specifically, features showing a peak rating > 7 in Compound Discoverer and a mzCloud match > 85% were assigned to level 2, extracted from the “cleaned‐up” feature list, and visualized using hierarchical clustering with the “Pheatmap” package in R‐Statistics.

### 4.6 Determination of Antioxidant Activity

#### 4.6.1 Radical Scavenging activity on ABTS•^+^ (2,2'‐Azino‐bis(3‐ethylben zothiazoline‐6‐sulfonic acid) and DPPH• (2,2‐diphenyl‐1 picrylhydrazyl)

The RSA on ABTS^•+^ radical was evaluated by the method described by Rodrigues et al. [75]. A stock solution of ABTS^•+^ (7.4 mM) was prepared in potassium persulfate (2.6 mM) for 16 h in the dark. The resulting ABTS•^+^ solution was diluted with ethanol to obtain an absorbance of approximately 0.7 at 734 nm. For the assay, samples (10 µL) at concentrations up to 10 mg/mL were mixed with 190 µl of ABTS•^+^ solution in 96‐well microplates, incubated for 6 min at RT, and the absorbance was measured at 734 nm on a microplate reader (Biochrom EZ Read 400, Santa Clara, CA, USA). The RSA on the DPPH^•^ radical was evaluated according to Rodrigues et al. [75]. Samples (22 µL at concentrations up to 10 mg/mL) were mixed with a DPPH solution (200 µL at 120 µM, in ethanol) and incubated for 30 min in darkness at RT. Absorbance was measured at 517 nm on a microplate reader (Biochrom EZ Read 400, Santa Clara, CA, USA). The RSA against ABTS•^+^ and DPPH• was expressed as the percentage of inhibition relative to a methanol control, and, when possible, as the half‐maximal effective concentration (EC_50_, mg/mL). Gallic acid (at concentrations up to 1 mg/mL) was used as a positive control.

#### 4.6.2 Metal Chelating Activity on Iron (ICA) and Copper (CCA)

The ICA and CCA were determined according to previously described protocols [75]. Briefly, for ICA, samples (30 µL; at concentrations up to 10 mg/mL) were mixed with distilled water (200 µL) and FeCl_2_ solution (30 µL; 0.1 mg/mL in water) in 96‐well plates. After an incubation of 30 min in the dark, ferrozine solution (12.5 µL; 40 mM in water) was added. The absorbance was measured at 562 nm on a microplate reader (Biochrom EZ Read 400, Santa Clara, CA, USA). For CCA determination, samples (30 µL, at concentrations up to 10 mg/mL) were mixed with Na acetate buffer (200 µL; 50 mM at pH 6), pyrocatechol violet (6 µL; 4 mM) in the above buffer, and CuSO4•5H_2_0 (100 µL; 50 g/mL in water). Absorbance was measured at 632 nm on a microplate reader (Biochrom EZ Read 400, Santa Clara, CA, USA). ICA and CCA results were expressed as a percentage of inhibition relative to a control containing methanol, and, when possible, as EC_50_ (mg/mL). Ethylenediaminetetraacetic acid (EDTA) was used as the positive control at concentrations up to 1 mg/mL.

#### 4.6.3 Ferric Reducing Antioxidant Power

FRAP was evaluated using the method described in Rodrigues et al. [75]. Samples (50 µL at concentrations up to 10 mg/mL) were mixed with distilled water (50 µL) and 1% potassium ferricyanide (50 µL) in 96‐well plates and incubated in the dark at 50°C for 20 min. Then, 10% trichloroacetic acid (50 µL) and 0.1% ferric chloride solution (50 µL) were added. Absorbance was measured at 700 nm on a microplate reader (Biochrom EZ Read 400, Santa Clara, CA, USA), and increased absorbance of the reaction means higher reducing power. Results were expressed as a percentage of inhibition relative to the positive control, butylated hydroxytoluene (BHT; 1 mg/mL), and as EC_50_ values, whenever possible.

### 4.7 Determination of the Cytotoxic Activity

Cytotoxicity was evaluated using four cell lines selected to provide a preliminary assessment of extract toxicity across tumor‐derived cells of different tissue origins and a nontumor‐derived reference model. The panel comprised human hepatocellular carcinoma HepG2 cells (RRID: CVCL_0027), human neuroblastoma SH‐SY5Y cells (RRID: CVCL_0019), murine melanoma B16 4A5 cells (RRID: CVCL_4612), and human embryonic kidney HEK293 cells (RRID: CVCL_0045), the latter being used as a nontumor‐derived reference model. HepG2 and SH‐SY5Y cell lines were kindly provided by the Biomedical Research Center (CBMR), University of Algarve (UAlg, Faro, Portugal), while UAlg provided HEK293 cells. The B16 4A5 cell line was purchased from Sigma‐Aldrich (Germany).

Cells were cultured in Dulbecco's modified Eagle medium (DMEM) supplemented with 10% heat‐inactivated fetal bovine serum (FBS), 1% L‐glutamine (final concentration of 2 mM), 50 U/mL penicillin, and 50 µg/mL streptomycin. All cell lines were maintained at 37°C in a humidified atmosphere containing 5% CO_2_.

For the cytotoxicity assay, exponentially growing cells were seeded in 96‐well culture plates at densities of 2 × 10^3^ cells/well for B16 4A5, 5 × 10^3^ cells/well for HepG2, and 1 × 10^4^ cells/well for SH‐SY5Y and HEK293. After a 24 h attachment period, the cells were exposed for 72 h to extracts diluted in supplemented DMEM at concentrations ranging from 0.98 to 125 µg/mL. Cell viability was assessed using the colorimetric 3‐(4,5‐dimethylthiazol‐2‐yl)‐2,5‐diphenyltetrazolium bromide (MTT) assay, as previously described by Rodrigues et al. [86]. Absorbance was measured at 590 nm using a Biochrom EZ Read 400 microplate reader (Biochrom Ltd., Cambridge, UK). Results were expressed as the percentage of cell viability relative to the vehicle control containing 0.5% dimethyl sulfoxide (DMSO). Concentration–response curves were used to calculate the half‐maximal inhibitory concentration (IC_50_), expressed in µg/mL.

### 4.8 Statistical Analysis

Experiments were performed at least in triplicate, and results are presented as mean ± standard error of the mean (SEM). Statistical analyses were conducted using XLSTAT for Microsoft Excel (Addinsoft 2024.2.0, New York, NY, USA). Student's *t*‐tests (*p* value < 0.05) were applied to compare total (poly)phenolics (TPC, TFC, F/F, HA, and CTC), mineral content, and for FRAP results. For the remaining antioxidant assays, significant differences were evaluated by one‐way analysis of variance (ANOVA) followed by Tukey's post hoc multiple comparison test (*p* < 0.05), assuming data normality. Curve fitting and EC_50_ calculations were performed using GraphPad Prism 9.5.1 (GraphPad Software, San Diego, CA, USA). Principal Component Analysis (PCA) was conducted using R‐Statistics software version 3.5.3.

## Author Contributions

Conceptualization: **Eliana Fernandes** and Luísa Custódio; Supervision: Luísa Custódio; Investigation: Eliana Fernandes, **Riccardo Trentin**; Methodology: Eliana Fernandes, Riccardo Trentin, Luísa Custódio; Formal analysis: Eliana Fernandes, Riccardo Trentin, **Maria João Rodrigues**, Luísa Custódio; Writing – Original draft preparation: Eliana Fernandes; Writing – Review & Editing: Eliana Fernandes and Luísa Custódio. All authors read and approved the final manuscript.

## Conflicts of Interest

The authors declare no conflicts of interest.

## Supporting information




**Supporting File 1**: cbdv71705‐sup‐0001‐SuppMat.docx

## Data Availability

The data that support the findings of this study are available on request from the corresponding author. The data are not publicly available due to privacy or ethical restrictions.
