# Supplementary material for: Contrasting Coastal Environments Are Associated With Ecotype‐Specific Leaf Polyphenolic Profiles and Bioactivity in Corema album subsp. album
Source: Chem Biodivers. 2026 Sep 20;23(9):e71705. doi: 10.1002/cbdv.71705 (PMC13590132; doi:10.1002/cbdv.71705)
Supplement: Supplementary file 1 [file CBDV-23-e71705-s001.docx]

Supplementary Material

**Figure S1-** Monthly meteorological conditions at the *Corema album* collection sites in Vila Real de Santo António (CL1) and Aljezur (CL2) from January 2021 to January 2022. (A) Mean maximum and minimum air temperature, (B) mean maximum and minimum relative humidity, and (C) cumulative global solar radiation (GSR) and precipitation. Temperature and relative humidity values are presented as monthly means ± SD. Meteorological data adapted from the Direção Regional de Agricultura e Pescas do Algarve [36].

**Table S1 -** Limits of detection (LOD) and quantification (LOQ), expressed as µg/g dw, for mineral determination in *Corema album* leaves by MP-AES.

| **Minerals** | **LOD** | **LOQ** |
| --- | --- | --- |
| **Ca** | 1,51 | 5,02 |
| **K** | 0,91 | 3,04 |
| **Mg** | 0,23 | 0,77 |
| **Na** | 0,47 | 1,57 |
| **Mn** | 0,01 | 0,01 |
| **Fe** | 0,83 | 2,77 |
| **Zn** | 0,11 | 0,36 |
| **Cu** | 0,19 | 0,62 |
| **Ni** | 0,12 | 0,39 |
| **Cr** | 0,01 | 0,03 |

**Table S2 -** Ultrahigh-Performance Liquid Chromatography Coupled with Heated Electrospray Ionization Mass Spectrometry (UHPLC–HESI–MS) putative identification of metabolites present in methanolic extracts of *Corema album* leaves ecotypes (CL1 and CL2).

| **ID** | **RT (min)** | **Proposed Structure** | **Proposed compound** | **m/z** | **Adduct** | **Adj_*p*-value** |
| --- | --- | --- | --- | --- | --- | --- |
| 1 | 0.93 | C₅H₁₃NO | Choline | 104.1066 | [M+H]^+^ | 0.011212 |
| 2 | 0.959 | C₇H₁₂O₆ | D-(-)-Quinic acid | 191.0562 | [M-H]^-^ | 0.017805 |
| 3 | 0.961 | C₁₂H₂₂O₁₁ | Sucrose | 377.0857 | [M+Cl]^-^ | 0.000896 |
| 4 | 1.021 | C₄H₆O₅ | L-(-)-Malic acid | 133.0146 | [M-H]^-^ | 0.025308 |
| 5 | 1.485 | C₆H₈O₇ | Citric acid | 191.0197 | [M-H]^-^ | 0.011311 |
| 6 | 6.167 | C₇H₆O₄ | Gentisic acid | 153.0195 | [M-H]^-^ | 0.004895 |
| 7 | 6.494 | C₁₃H₁₆O₉ | 2-hydroxy-5-[3,4,5-trihydroxy-6-(hydroxymethyl)oxan-2-yl]oxybenzoic acid | 315.0719 | [M-H]^-^ | 0.128178 |
| 8 | 8.261 | C₇H₆O₃ | 4-Hydroxybenzoic acid | 137.0247 | [M-H]^-^ | 0.104335 |
| 9 | 9.339 | C₉H₈O₃ | 2-Hydroxycinnamic acid | 163.0403 | [M-H]^-^ | 0.016182 |
| 10 | 10.258 | C₁₀H₁₀O₄ | Ferulic acid | 193.0506 | [M-H]^-^ | 0.116172 |
| 11 | 10.469 | C₁₅H₂₀O₈ | 4-Acetyl-3-hydroxy-5-methylphenyl β-D-glucopyranoside | 327.1083 | [M-H]^-^ | 0.01804 |
| 12 | 11.756 | C₂₁H₂₂O₁₁ | 3,5-Dihydroxy-2-(4-hydroxyphenyl)-4-oxo-3,4-dihydro-2H-chromen-7-yl hexopyranoside | 449.1092 | [M-H]^-^ | 0.23769 |
| 13 | 11.93 | C₁₅H₁₀O₈ | Myricetin | 319.0445 | [M+H]^+^ | 0.056016 |
| 14 | 11.931 | C₂₁H₂₀O₁₃ | myricetin 3-O-beta-D-galactopyranoside | 481.0969 | [M+H]^+^ | 0.06135 |
|  | 11.946 |  |  | 479.0828 | [M-H]^-^ | 0.330937 |
| 15 | 12.074 | C₈H₈O₄ | 3-Methoxysalicylic acid | 167.0351 | [M-H]^-^ | 0.077639 |
| 16 | 12.822 | C₁₅H₁₀O₇ | 2-(2,6-dihydroxyphenyl)-3,5,7-trihydroxy-4H-chromen-4-one | 303.0497 | [M+H]^+^ | 0.014481 |
| 17 | 13.998 | C₂₁H₂₀O₁₂ | Quercetin-3β-D-glucoside | 465.102 | [M+H]^+^ | 0.201507 |
|  | 14.056 |  |  | 463.0879 | [M-H]^-^ | 0.001182 |
| 18 | 14.186 | C₁₅H₁₀O₆ | Fisetin | 287.0546 | [M+H]^+^ | 0.002506 |
| 19 | 14.597 | C₂₂H₂₂O₁₂ | 5,7-dihydroxy-2-(4-hydroxy-3-methoxyphenyl)-3-{[3,4,5-trihydroxy-6-(hydroxymethyl)oxan-2-yl]oxy}-4H-chromen-4-one | 479.1174 | [M+H]^+^ | 0.000868 |
| 20 | 14.624 | C₁₆H₁₂O₇ | 3-Methoxy-5,7,3',4'-tetrahydroxy-flavone | 317.0652 | [M+H]^+^ | 0.001058 |
| 21 | 14.663 | C₂₀H₁₈O₁₁ | Reynoutrin | 435.0913 | [M+H]^+^ | 0.001046 |
| 22 | 14.918 | C₂₁H₂₀O₁₁ | Kuromanin | 449.107 | [M+H]^+^ | 0.479088 |
| 23 | 14.918 | C₁₅H₁₀O₆ | Kaempferol | 287.0547 | [M+H]^+^ | 0.944259 |
| 24 | 14.934 | C₂₁H₂₀O₁₁ | Trifolin | 447.093 | [M-H]^-^ | 0.003219 |
| 25 | 15.336 | C₂₁H₂₂O₁₀ | 7-Hydroxy-2-(4-hydroxyphenyl)-4-oxo-3,4-dihydro-2H-chromen-5-yl β-D-glucopyranoside | 433.1137 | [M-H]^-^ | 0.82039 |
| 26 | 17.1 | C₁₀H₈O₄ | 7,8-Dihydroxy-4-methylcoumarin | 191.0348 | [M-H]^-^ | 0.004328 |
| 27 | 17.766 | C₁₅H₁₀O₇ | Quercetin | 301.035 | [M-H]^-^ | 0.003446 |
| 28 | 17.995 | C₁₅H₁₄O₅ | Phloretin | 273.0765 | [M-H]^-^ | 0.336872 |
| 29 | 19.02 | C₁₈H₃₂O₅ | Corchorifatty acid F | 327.2172 | [M-H]^-^ | 0.000488 |
| 30 | 19.947 | C₁₅H₁₀O₅ | Apigenin | 269.0445 | [M-H]^-^ | 0.88966 |
| 31 | 20.309 | C₁₂H₂₇NO | N,N-Dimethyldecylamine N-oxide | 202.2162 | [M+H]^+^ | 0.071534 |
| 32 | 20.324 | C₁₅H₁₀O₃ | 5-hydroxy-2-phenyl-4H-chromen-4-one | 239.07 | [M+H]^+^ | 0.0381 |
| 33 | 21.017 | C₁₆H₁₄O₅ | Sakuranetin | 287.0911 | [M+H]^+^ | 0.206383 |
|  | 21.029 |  |  | 285.0765 | [M-H]^-^ | 0.009699 |
| 34 | 21.666 | C₁₇H₁₆O₅ | 2,2′-Dihydroxy-4′,6′-dimethoxychalcone | 301.1068 | [M+H]^+^ | 0.412468 |
| 35 | 22.265 | C₁₅H₁₀O₄ | Chrysin | 255.0654 | [M+H]^+^ | 0.581914 |
| 36 | 22.325 | C₁₅H₁₀O₄ | Rubiadin | 253.0516 | [M-H]^-^ | 0.122382 |
| 37 | 22.853 | C₃₀H₄₈O₅ | Asiatic acid | 487.342 | [M-H]^-^ | 0.192326 |
| 38 | 23.448 | C₁₆H₁₄O₄ | (2R)-5-hydroxy-7-methoxy-2-phenyl-3,4-dihydro-2H-1-benzopyran-4-one | 271.0962 | [M+H]^+^ | 0.113099 |
| 39 | 23.516 | C₂₄H₃₀O₆ | Bis(4-ethylbenzylidene)sorbitol | 415.2106 | [M+H]^+^ | 0.162609 |
| 40 | 24.123 | C₁₆H₁₆O₄ | 2',6'-Dihydroxy 4'-methoxydihydrochalcone | 273.1118 | [M+H]^+^ | 0.075458 |
| 41 | 25.378 | C₁₅H₁₂O₅ | Naringeninchalcone | 273.0755 | [M+H]^+^ | 0.140123 |
| 42 | 25.926 | C₁₆H₁₄O₄ | Cardamomin | 271.0963 | [M+H]^+^ | 0.017915 |
| 43 | 26.09 | C₃₀H₄₆O₄ | 18-β-Glycyrrhetinic acid | 471.3458 | [M+H]^+^ | 0.966103 |
| 44 | 26.31 | C₁₈H₃₇NO | Stearamide | 284.2943 | [M+H]^+^ | 0.843125 |
| 45 | 26.616 | C₃₀H₄₈O₄ | Hederagenin | 471.3475 | [M-H]^-^ | 0.6453 |
| 46 | 28.047 | C₁₅H₁₂O₄ | Pinocembrin | 257.0807 | [M+H]^+^ | 0.020411 |
| 47 | 29.369 | C₁₅H₁₀O₄ | 5,7-dihydroxy-2-phenyl-4H-chromen-4-one | 255.065 | [M+H]^+^ | 0.083396 |
| 48 | 29.444 | C₁₆H₁₄O₄ | 5-Hydroxy-7-methoxyflavanone | 271.0962 | [M+H]^+^ | 0.004683 |
| 49 | 30.332 | C₃₀H₄₈O₃ | Oleanolic acid | 457.3665 | [M+H]^+^ | 0.228985 |
|  | 30.359 |  |  | 455.3524 | [M-H]^-^ | 0.25103 |

RT - retention times (minutes).
